# Glue Which Will Unite Even Polished Steel

**Published:** 1869-07

**Authors:** 


Glue which will Unite even Polished Steel.—A Turkish receipt for a cement used to fasten diamonds and other precious stones to metallic surfaces, and which is said to strongly unite even surfaces of polished steel, although exposed to moisture, is as follows:

"Dissolve five or six bits of gum mastic, each of the size of a large pea, in as much spirits of wine as will suffice to render it liquid. In another vessel, dissolve in brandy as much isinglass, previously softened in water, as will make a two ounce vial of strong glue, adding two small bits of gum ammoniac, which must be rubbed until dissolved. Then mix the whole with heat. Keep in a vial closely stopped. When it is to be used, set the vial in boiling water.

